# The Shepard Illusion Is Reduced in Children With an Autism Spectrum Disorder Because of Perceptual Rather Than Attentional Mechanisms

**DOI:** 10.3389/fpsyg.2018.02452

**Published:** 2018-12-04

**Authors:** Philippe A. Chouinard, Kayla A. Royals, Oriane Landry, Irene Sperandio

**Affiliations:** ^1^Department of Psychology and Counselling, School of Psychology and Public Health, La Trobe University, Melbourne, VIC, Australia; ^2^School of Psychology, University of East Anglia, Norwich, United Kingdom

**Keywords:** autism spectrum disorder, ASD, visual perception, Shepard illusion, eye-tracking

## Abstract

Earlier studies demonstrate reduced illusion strength in the Shepard illusion in adults and adolescents with an autism spectrum disorder (ASD) and in typically developing (TD) adults with high levels of autistic traits. We measured the strength of the Shepard illusion in ASD and TD children and tested if ten different eye-tracking measurements could predict group differences in illusion strength. The ASD children demonstrated reduced illusion strength relative to the TD group. Despite this, there were no mean differences on any of the eye-tracking measurements between groups. Even though none of the eye-tracking measurements revealed mean differences between the two groups, the degree to which spatial attention was directed toward the standard stimulus, as indexed by the number of saccades within and toward this stimulus, predicted the strength of the illusion in the overall sample. Furthermore, this active scanning of the standard stimulus was found to enhance illusion strength more strongly in the ASD than the TD group. Together, we conclude that scan patterns and the degree to which participants are able to shift between different locations in a visual scene did not account for group differences in illusion strength. Thus, the reduced strength of the Shepard illusion in ASD does not appear to be driven by how attention shifts or is spatially allocated. Rather, differences may relate instead to perceptual mechanisms that integrate visual information. Strategies that may aid ASD individuals to see this illusion more strongly could have them make even more eye movements within and between the stimuli presented in the illusion display.

## Introduction

Atypical perception is not included among the diagnostic criteria for autism spectrum disorder (ASD). Yet, there is an abundance of evidence that children with ASD manifest perceptual styles that differ from other children. These observations have led to a number of different theories. For example, the Enhanced Perceptual Functioning (EPF) theory (Mottron et al., 2006) proposes that perception is more veridical and detailed-focussed in children with ASD and that these differences play a causal role in many of its symptoms. In contrast, the Weak Central Coherence (WCC) theory proposes that these same perceptual differences are a consequence of a weakened tendency to process and integrate information globally (Happé and Frith, 2006). More recently, Bayesian explanations have been proposed (Pellicano and Burr, 2012; van Boxtel and Lu, 2013; Lawson et al., 2014; van de Cruys et al., 2014; Davis and Plaisted-Grant, 2015). According to these explanations, the child’s past experiences, or priors, are important in shaping perception (Helmholtz, 1867; Gregory, 1980) and the use of priors in persons with ASD is attenuated. Specifically, the active process of formulating and testing hypotheses about the world is reduced, which results in greater immunity to suggestion and a tendency to perceive the world more as it really is.

Illusions offer researchers the opportunity to determine if this is indeed the case. Most illusions are driven by mechanisms that are generally helpful for perception but trick us into perceptually rescaling sensory information in a manner that is unnecessary or incorrect in a given context, leading to changes in perception that differ from what is physically present (Gregory, 2015). According to the above theories, individuals with ASD should perceive weaker illusions than typically developing (TD) children. The EPF and WCC theories predict that ASD individuals are less sensitive to the misleading global contextual cues that give rise to many visual illusions, resulting in reduced levels of susceptibility to them. Meanwhile, Bayesian interpretations predict that individuals with ASD would be less susceptible to illusions in general, because they are less influenced by prior experiences and knowledge about the world (Pellicano and Burr, 2012).

A considerable amount of research has been carried out examining visual illusions in individuals with ASD. However, results from these studies are mixed (Van der Hallen et al., 2015). While the first research by Happé (1996) concluded that children with ASD are less susceptible to most visual illusions, the majority of studies since this first study have demonstrated that ASD individuals are just as susceptible to most visual illusions as TD individuals (Ropar and Mitchell, 1999, 2001; Hoy et al., 2004; Rouse et al., 2004; Schwarzkopf et al., 2014; Manning et al., 2017). It is unclear to what extent the heterogeneity in sampling characteristics may have contributed to these inconsistencies, which is why other studies have re-examined the strength of various visual illusions as a function of autistic traits in the TD population (Walter et al., 2009; Chouinard et al., 2013, 2016). Such an approach serves to model ASD while reducing confounds related to differences in symptom severity, cognitive ability, development, and co-morbid disorders (Landry and Chouinard, 2016).

**FIGURE 1:**
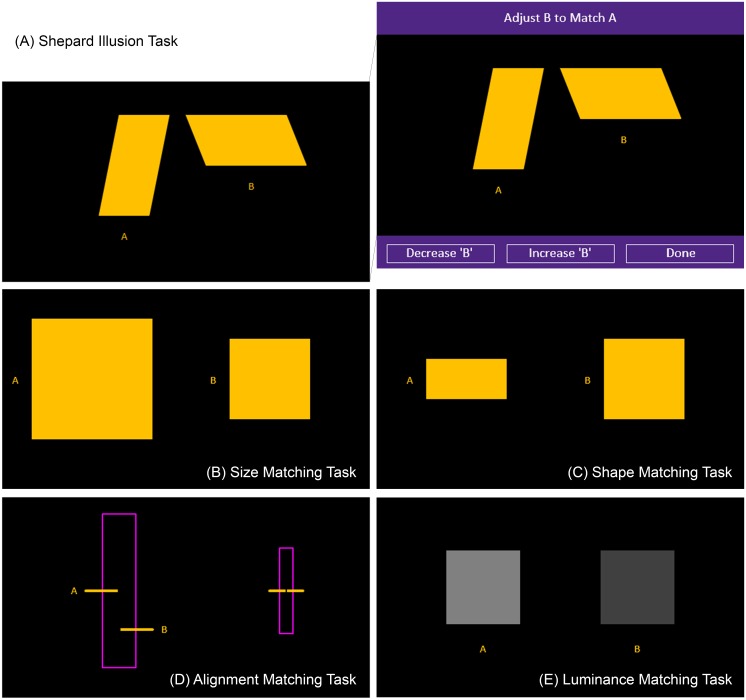
Illusion and control matching tasks. The figure displays the illusion and control matching tasks that were examined. The illusion task consisted of the Shepard illusion **(A)**. The right side of the top panel **(A)** demonstrates the full visual display with the buttons at the bottom that the participants used to adjust the comparison stimulus (in this case, the stimulus on the right) to match the standard (in this case, the stimulus on the left). The different tasks **(A–E)** had similar buttons and differed by the stimuli presented in the black area and the dimensions that had to be matched. The control tasks consisted of size **(B)**, shape **(C)**, alignment **(D)**, and luminance **(E)** matching tasks.

In the most recent of these studies, Chouinard et al. (2016) presented a battery of thirteen visual illusions to participants, and demonstrated that only two were correlated with autistic traits as measured by the autism quotient (AQ) (Baron-Cohen et al., 2001) questionnaire: the Shepard and square-diamond illusions. The Shepard illusion is a powerful illusion in which a parallelogram appears to have a different height and width when it is rotated 90 degrees (Figure 1A; Shepard, 1990). The square-diamond illusion is a variant of the Shepard illusion in which a square appears smaller when it is rotated 45 degrees and presented as a diamond. Chouinard et al. (2016) demonstrated a 22 and 5% perceptual difference in size between orientations in the Shepard and square-diamond illusions, respectively. In addition, the magnitude of these perceptual differences decreased as the level of autistic traits increased. These findings converge well with Mitchell et al. (2010), who demonstrated attenuation in the strength of the Shepard illusion in a group consisting mostly of adolescents and adults with ASD. Specifically, their sample consisted of ASD participants between the ages of 12 to 29 years (*M* = 21.1, *SD* = 5.0).

Given only two out of thirteen illusions demonstrated a relationship with autistic traits, Chouinard et al. (2016) questioned the EPF, WCC, and Bayesian theories of perception in ASD and proposed instead that perceptual styles in ASD might relate to differences in specific types of high-level visual integration, such as those implicated in shape processing or mental rotation, as opposed to a style that privileges local details over global integration or depends on atypical priors in an all-encompassing manner. In addition, Chouinard et al. (2013, 2016) elaborated further that perceptual differences in ASD might be specific to the processing of contextual elements embedded within stimuli, which is the case for the Shepard illusion, as opposed to between stimuli, which is the case for a number of illusions, including the well-known Ebbinghaus illusion (Titchener, 1901). Chouinard et al. (2016) argued that their results could generalize to ASD given that Mitchell et al. (2010) had previously demonstrated similar effects with the Shepard illusion in an adolescent/adult population with ASD. Whether or not similar results could be demonstrated in children with ASD has never been investigated until now.

There is an alternative explanation that Chouinard et al. (2016) and all other previous studies on illusions in ASD have not considered before. Perhaps it could be the case that differences in how spatial attention shifts and is allocated to different parts of a visual scene might be the underlying cause for decreases in illusion strength. It is conceivable that illusions, which depend on the integration of visual information, may be weaker in ASD because these individuals either manifest reduced abilities in shifting attention or attend to different parts of the visual scene. In the first case, ASD individuals may not see the illusion as strongly because their eyes move between different locations in the visual scene differently. In the second case, TD individuals might direct their attention to certain parts of the display that are more important to drive the illusion because they find the percept itself more interesting, which causes the illusion to be stronger.

For these reasons, we considered a number of eye-tracking measurements. The first series of measurements, consisting of *saccade velocity* and *saccade frequency*, served to index abilities in shifting attention (Schmitt et al., 2014). We reasoned that persons with ASD would demonstrate slower and less frequent saccades relative to TD individuals if their abilities to disengage and direct their attention to different parts of a visual scene were reduced. Indeed, a meta-analysis by Landry and Parker (2013) demonstrated a consensus in the literature for diminished performance in ASD on the Posner endogenous cueing task, which gages goal-directed shifts in attention. It has been further suggested that these impairments in shifting attention might play an important role in atypical perception (Laycock et al., 2007) and other symptoms (Laycock et al., 2007; Landry et al., 2009) in ASD. If this is the case then one might expect to find differences between ASD and TD groups on these measurements and that they might correlate with illusion strength.

The next series of measurements arose from a spatial analysis of where participants attended to in the visual scene. The stimuli in the Shepard illusion consisted of two parallelograms. We calculated the proportion of saccades that were made within each shape, between them, and elsewhere. We had no specific predictions as to how the allocation of spatial attention might affect the strength of the illusion given this is the first eye tracking study on the illusion that we know of. The last measurement, *completion times*, served as an index of task engagement. This measurement was important to consider so we could rule out the possibility that differences in illusion strength were due to differences in task engagement.

The present study aimed to determine if the strength of the Shepard illusion is diminished in ASD relative to TD children and whether or not this reduction, if present, can be explained by differences in shifts in attention or where attention is spatially allocated in the visual scene. To this end, the Shepard illusion was examined in ASD and TD children while an eye-tracker recorded eye movements. It was hypothesized that the children with ASD would demonstrate reduced illusion strength to the Shepard illusion relative to the TD children, converging with previous findings in an older sample with ASD (Mitchell et al., 2010) and TD adults with various degrees of autistic traits (Chouinard et al., 2016). It was also hypothesized that the group difference in illusion strength, if present, could be explained by eye-tracking measurements.

## Materials and Methods

### Participants

Our sample consisted of 18 individuals with ASD (12 males, age range 6.5 – 15.5, mean = 11.4) and 18 TD individuals (12 males, age range 6.0 – 14.7, mean = 11.4) (Table 1). Originally, we collected data from 23 ASD participants. Two were excluded from analyses for not completing the task and three were excluded from analyses due to insufficient eye-tracking data collection (eye positioning could only be recorded less than 20% of the time). We recruited the ASD participants from a summer program for children with learning disabilities at a major Australian city (Melbourne, VIC). All participants attended mainstream schools during the school year. The ASD participants had been diagnosed in accordance with the Diagnostic and Statistical Manual of Mental Disorders IV Text Revision (DSM-IV-TR) (American Psychiatric Association, 2000) by an independent multidisciplinary team, which included the use of the Autism Diagnostic Observation Schedule (ADOS) (Lord et al., 1989) and Autism Diagnostic Interview (ADI) (Lord et al., 1994).

**Table 1:** Gender, age, and raw RPM scores for the ASD and TD groups.

	*ASD*	*TD*

*N*	12 Males 6 Females	12 Males 6 Females
*Age*		
*Mean (SD)*	11.38 (2.86)	11.38 (2.52)
*Range*	6.5 – 15.5	6.0 – 14.7
RPM raw score		
*Mean (SD)*	34.72 (11.79)	34.89 (11.09)
*Range*	9 – 50	14 – 51

*Both groups were matched for gender, age, and raw scores on the RPM*.

The 18 TD individuals were selected from a sample of more than 100 children and adolescents from a regional Australian city (Bendigo, VIC), who completed the same tests as part of a larger study (not yet published). Those who were best matched to the ASD participants on age, gender, and raw scores on the Raven Progressive Matrices (RPM) (Raven et al., 2003) were selected as controls for this study. The person selecting these participants (OL) was blind to how the ASD and TD participants performed on all other tasks. For screening purposes, parents of the TD participants were asked to indicate if their child had a diagnosis of ASD or any other neurological or psychiatric condition. All children in the final sample had normal or corrected-to-normal vision and demonstrated good compliance.

Written informed consent was obtained from a parent or legal guardian and written assent was obtained from the child participants. This research was approved by the Australian State of Victoria’s Department of Education and Training and the La Trobe University Human Ethics Committee in accordance with the Declaration of Helsinki.

### Procedures

We used the Tobii Pro TX300 (Tobii AB, Danderyd, Sweden) system connected to a Dell Precision M6800 mobile workstation (Dell Inc, Round Rock, TX, United States) to record eye positioning in Cartesian *X, Y* coordinates. The screen monitor of this system was 23 inches with a resolution of 1920 × 1080 pixels. The system captured 300 frames per second and coded each frame as either a fixation or a saccade. The raw eye-tracking data was then analyzed in Matlab (Mathworks, Natick, MA, United States) using in-house scripts.

Eye tracking began with a brief calibration procedure, which was always carried out with the participant’s head placed on a chin rest at a viewing distance of 60 cm. During calibration, the participant was asked to track with their eyes a red circle that moved between nine positions on the computer screen (top left, center and right; middle left, center and right; bottom left, center and right). After this registration, the system recorded the data in *X, Y* Cartesian coordinates relative to the top left corner of the computer screen in pixels. Following calibration, the participant completed a series of control tasks (size matching, shape matching, alignment matching, and luminance matching), and the Shepard illusion task. All participants performed the control tasks before the illusion task. Half the participants completed the RPM before the control and illusion tasks while the other half did it afterward – the order being randomly assigned for each individual. The control and illusion tasks were programmed in Action Script (Adobe Systems, San Jose, CA, United States) and presented using Flash player (Adobe Systems, San Jose, CA, United States). The RPM was carried out without eye-tracking as this task was not computerized.

For both the control and illusion tasks, the participant had to adjust a comparison stimulus to appear the same along a physical dimension as a standard stimulus by pressing the *Decrease* and *Increase* buttons displayed on the bottom-left and bottom-center of the computer screen (right panel in Figure 1A). The participants were given as much time as they needed to complete each trial and were asked to press the *Done* button displayed on the bottom-right of the computer screen when they felt they had matched the comparison stimulus to the standard one. The participants completed one trial for each of the control matching tasks and four trials for the illusion task. The order of the control matching tasks was generated randomly for each participant. All displays had a black background.

The participants were encouraged to base their adjustments on how the stimuli appeared. At the start of each task, the experimenter would say something along these lines: *“In this activity, we will be matching different shapes. We want you to make the two [experimenter would say and point to what features needed be match] appear the same. You’re going to be make this one [experimenter points to the comparison] smaller or larger so it looks like that one [experimenter points to the standard]. This button makes it larger [experimenter points to appropriate button] and this button makes it smaller [experimenter points to appropriate button]. Once you’re happy that the two look the same [experimenter would again say and point to what features needed be matched], press this button [experimenter points to the Done button].”* Slight variations in the instructions were made to the alignment and luminance matching tasks given that they entailed matching for orientation and brightness, as opposed to size, respectively. Further clarification was provided when required.

In the size matching control task, which assessed size discrimination, the display consisted of two yellow squares (Figure 1B). The square on the right was designated as the standard, which remained fixed at 120 pixels in length, while the square on the left was designated as the comparison stimulus, which the participant adjusted. The size of the comparison stimulus began at 180 pixels in length. Scores were obtained by calculating the final difference in pixels between the fixed length of the standard and the adjusted length of the comparison stimulus.

In the shape matching control task, which assessed shape discrimination, the display consisted of two yellow four-sided shapes (Figure 1C). The rectangle on the left was designated as the comparison stimulus while the square on the right was designated as the standard stimulus. The height and width of the standard remained fixed at 120 pixels. The width of the comparison remained fixed at 120 pixels while the height was adjusted by the participants so that it matched the standard. The height of the comparison stimulus began at 60 pixels. Scores were obtained by calculating the final difference in pixels between the fixed height of the standard and the adjusted height of the comparison stimulus.

In the alignment matching control task, which assessed abilities in Verner acuity, the display consisted of two horizontal yellow lines passing perpendicularly through the long axis of a rectangle outlined in magenta, which was presented in the upright position (Figure 1D). The yellow line on the left served as the standard while the line on the right served as the comparison, which was initially presented 57 pixels lower than the standard. The participant’s task was to align the comparison stimulus to match the standard. Scores were obtained by calculating the final difference in pixels between the fixed vertical position of the standard and the adjusted vertical position of the comparison stimulus.

In the luminance matching control task, which assessed luminance discrimination, the display consisted of two gray squares (Figure 1E). The one on the left had an RGB value of [128, 128, 128]. This square was the standard. The other, which served as the comparison stimulus, was presented with an initial RGB value of [64, 64, 64]. Both squares were 110 pixels wide. The participant’s task was to adjust the luminance of the comparison stimulus to match the standard. Scores were obtained by calculating the final difference in RGB values between the fixed luminance of the standard and the adjusted luminance of the comparison stimulus.

The Shepard illusion task consisted of two yellow parallelograms (Figure 1A). The parallelogram on the left was oriented vertically while the one on the right was oriented horizontally. On each trial, one of the parallelograms was designated as the standard while the other was designated as the comparison stimulus. The comparison stimulus was initially presented either 50% smaller or 50% bigger than the standard. The order of the trials, each representing one of the four possible starting combinations, was generated randomly. The length of both parallelograms remained fixed at 180 pixels. The width of the standard remained fixed at 75 pixels while the width of the comparison stimulus was adjusted by the participants so that it matched the standard. The apparent width of the parallelogram on the left was typically smaller than the one on the right when both were physically identical.

The participants also completed the RPM, which is a non-verbal measure of general cognitive ability. In this assessment, the participant was provided with a booklet of different patterns, with a piece missing in each pattern. For each item, the participant was required to select which piece best matched the pattern. Depending on the age of the child, this assessment usually took between 15 and 30 min to complete. There were two forms of the RPM, designed for different age groups. The colored version was administered to children younger than 10 years, while the standard version was administered to children over the age of 10 years. Scores on the colored form were converted to standard scores based on conversion tables in the RPM manual.

### Data Analyses

The data were analyzed using JASP software version 0.8 (University of Amsterdam, Amsterdam, Netherlands) and GraphPad Prism version 7 (GraphPad Software Inc., La Jolla, CA, United States). Given it is well accepted that some illusions are stronger than others, perceptual measurements from the illusion task were normalized to allow for meaningful comparisons between this study and other studies that have also used normalized susceptibility measures (Schwarzkopf et al., 2011; Chouinard et al., 2013, 2017, 2016). Specifically, normalized indices of illusion strength were calculated as follows: [(Perceived Size in Configuration A - Perceived Size in Configuration B)/(Perceived Size in Configuration A + Perceived Size in Configuration B); configuration A denoting the condition one would expect to see greater judgements in perceived size]. Based on this formula, positive values denote perceptual effects in the expected direction for the illusion while negative values denote perceptual effects in the opposite direction.

One-sample *t*-tests against zero were used to measure illusion strength in each group. In cases when normality could be assumed according to Shapiro-Wilk tests, independent samples *t*-tests were used to investigate differences between groups in illusory strength as well as performance on the control tasks. Otherwise, Mann-Whitney tests were performed. In the tables, we report both uncorrected *p*-values as well as corrected *p*-values for the number of control tasks examined, using the Bonferroni method (i.e., *p_corr_* = *p_uncorr_* × 4). Similar corrections were not applied to the illusion task given we had a clear *a priori* prediction based on previous investigations of similar tasks performed in an adolescent/adult population with ASD (Mitchell et al., 2010) and a typically developing adult population that varied in autistic traits (Chouinard et al., 2016). *Cohen’s d* is reported for illusion strength.

In addition to null hypothesis statistical testing (NHST), which does not allow one to draw definite conclusions about the viability of the null hypothesis, we calculated Bayes Factors (*BF_10_*) denoting the likelihood of the alternative over the null hypothesis. Within the framework of Bayesian statistics, one quantifies the evidence in support for either the alternative or the null hypothesis relative to the other (Wagenmakers, 2007). A *BF_10_* value of 3 or more was considered to provide substantial support for the alternative hypothesis and values less than 0.333 to provide substantial support for the null hypothesis (Jeffreys, 1961). There is no need to correct for multiple Bayes factors given that they do not reflect probabilities (Gelman et al., 2012). The Bayes analyses allowed us to determine if a different statistical approach might converge with NHST, which would provide more confidence in the findings, and draw more definite inferences from null results, which NHST is not designed to evaluate.

We calculated the following dependent variables from the eye-tracking data in the Shepard illusion task that were confined within the black viewing area shown in Figure 1. *Saccade velocity* was calculated by taking the Euclidian distance in pixels between the *X, Y* coordinates before and after the saccade and dividing this number by the duration of the saccade in milliseconds, expressing the measurement in pixels by milliseconds. *Saccade frequency* was calculated by the number of saccades registered by the Tobii system during data collection divided by total duration in minutes, expressing the measurement in number of saccades per minute. *Completion time* was calculated as the amount of time in seconds to complete a trial.

We also performed a detailed spatial analysis of the eye-tracking data. This was carried out by an experienced research assistant in the lab (KAR) who was blind to participant identity and group membership. Given the dynamic and self-paced nature of the visual stimuli in which the comparison stimulus changed during task performance, the research assistant played back the eye-tracking recordings in Tobii Pro Studio (Tobii AB, Danderyd, Sweden) and manually classified each saccade movement as either *standard-to-standard, comparison-to-comparison, standard-to-comparison, comparison-to-standard*, or *other*. We express each of these measurements as a percentage of the total number of saccades made.

We also examined how much time participants spent fixating on the two stimuli. Two areas-of-interest (AOI) were created over the standard and comparison stimuli. Each was equal in area to the largest presentation of the comparison stimulus. From this, we calculated looking time for each AOI as a percentage of the total time spent fixating somewhere inside the boundaries of the black viewing area shown in Figure 1. This was carried out for both *time spent fixating on the standard stimulus* and *time spent fixating on the comparison stimulus.* We reasoned that more saccades to and/or more time spent on the standard might mean that the participant is concentrating more on the stimulus that they are being asked to base their perceptual judgements, while more saccades to and/or more time on the comparison might mean that the participant is concentrating more on the stimulus that they are being asked to adjust – detracting their attention away from the standard stimulus.

The eye-tracking data also underwent NHST and Bayesian analyses. Independent samples *t*-tests were used to evaluate differences between groups and Pearson correlations were used to examine associations between illusory strength and each of the eye-tracking dependent variables across groups. In the tables, we report both uncorrected and corrected *p*-values adjusted for multiple comparisons using the Bonferroni method (*p_corr_* = *p_uncorr_* × 10). Corresponding Bayes factors (*BF_10_*) are also reported. As mentioned previously, there is no need to correct for multiple Bayes factors (Gelman et al., 2012). Additional analyses were performed in certain cases to understand further significant effects (or lack of) obtained from the above analyses. Each of these extra tests is described in the Results section. Effects obtained from all analyses were deemed significant at an alpha of 0.05.

## Results

Independent samples *t*-tests demonstrated that there were no differences in age (*t*_(34)_ = 0.009, *p* = 0.993, *BF_10_* = 0.322) and raw RPM scores (*t*_(34)_ = 0.044, *p* = 0.965, *BF_10_* = 0.322) between the ASD and TD groups (for means and other descriptive statistics, see Table 1). The male to female ratio was the same for both groups. Thus, the two groups were matched for age, gender, and raw RPM scores.

### Performance on the Control Tasks

An independent samples *t*-test demonstrated that performance on the luminance task between the ASD and TD groups did not differ (*p* = 0.341), while Mann-Whitney tests also revealed no differences between the two groups on the size, shape, and alignment tasks (all *p* ≥ 0.341; for more detailed statistics, see Table 2). Bayesian analysis further demonstrated substantial support for the null relative to alternative hypothesis in the shape matching task (*BF_10_* = 0.332) (Table 2). All other *BF_10_* ranged from 0.358 to 0.463 (Table 2). Although none of the other *BF_10_* values were indicative of substantial support for the null hypothesis, they were still all below 1 – demonstrating a tendency for more support for the null than the alternative hypothesis. Thus, the two groups seemed to not differ on the various perceptual discrimination abilities tested.

**Table 2:** Performance on the control tasks in the ASD and TD groups.

	*Mean ASD (SD)*	*Mean TD (SD)*	*t_(34)_*	*U*	*p_uncorr_*	*p_corr_*	*BF_10_*
*Size matching (pixel difference)*	4.94 (7.88)	3.58 (3.30)	-	131.50	0.341	1	0.385
*Shape matching (pixel difference)*	-0.94 (4.25)	-0.58 (3.30)	-	153.50	0.800	1	0.332
*Alignment matching (pixel difference)*	0.19 (0.79)	0.08 (0.43)	-	163.00	0.983	1	0.358
*Luminance matching (RGB increment difference)*	-4.75 (13.18)	-1.14 (8.80)	0.97	-	0.341	1	0.463

*p_corr_-values reported in column 7 were corrected for multiple comparisons using the Bonferroni method. Either independent samples *t-*tests (column 4) or Mann-Whitney tests (column 5) were performed depending on whether or not normality could be assumed as determined by a Shapiro-Wilk test*.

### Strength of the Shepard Illusion

**FIGURE 2:**
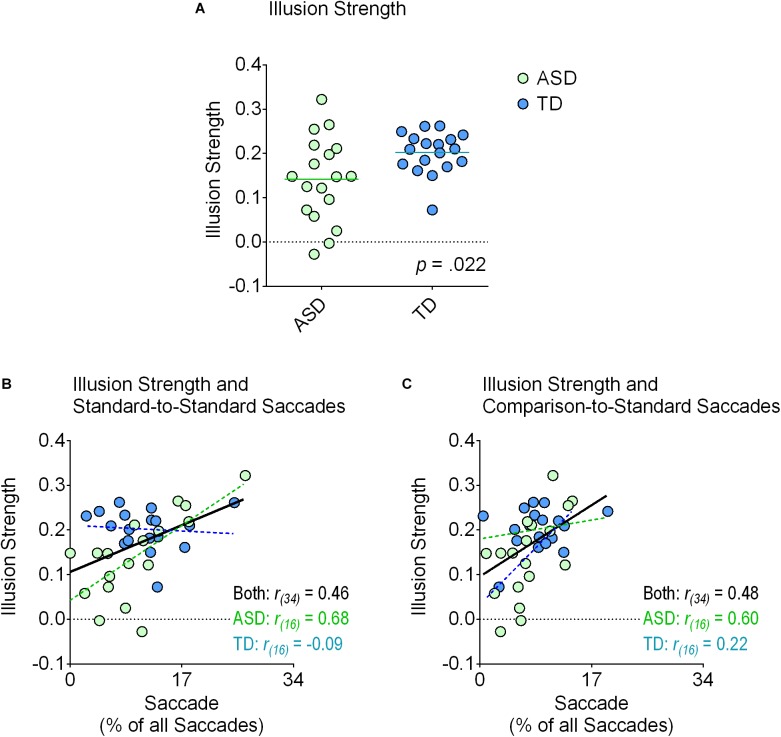
Illusion strength and correlations with saccadic eye movements to the standard stimulus. The top panel **(A)** displays each individual’s susceptibility index score (green circles, ASD group; blue circles, TD group) for the Shepard illusion. The lines represent the means for each group. As shown in the graph, we found a significant reduction in the ASD relative to the TD group. The other plots demonstrate the strength of the Shepard illusion as a function of the percentage of saccades that were classified as starting from one location to a different location within the standard stimulus **(B)** and classified as starting from one location on the comparison stimulus to a location on the standard stimulus **(C)**. In both instances, the overall correlations (in black) were significant after correcting for multiple comparisons using the Bonferroni method (*p* < 0.05). Additional linear regression analyses revealed that these correlations were primarily driven by the ASD group. Specifically, in both cases, the slopes between the two groups were different from each other (*p* < 0.05), positively correlated in the ASD group (*p* < 0.05; in green), and not correlated at all in the TD group (*p* ≥ 0.378; in blue).

All participants in the TD group demonstrated a positive susceptibility index score, indicating that they experienced the illusion in the expected direction (Figure 2A). In the ASD group, 16 participants had a positive score while 2 participants had a negative score (Figure 2A). One-sample *t*-tests demonstrated that illusion strength was greater than zero in both the ASD (*t_(17)_* = 6.35, *p* < 0.001, *Cohen’s d* = 1.50, *BF_10_* = 2,936) and TD (*t_(17)_* = 18.32, *p* < 0.001, *Cohen’s d* = 4.32, *BF_10_* > 10,000) groups. Independent samples *t*-tests revealed that the ASD group experienced a weaker illusion than the TD group (*t_(34)_* = 2.41, *p* = 0.022, *Cohen’s d* = 0.80, *BF_10_* = 2.81). In short, both groups experienced seeing the illusion but the ASD group experienced seeing a weaker one.

### Eye Tracking

There were no differences between the ASD and TD groups on any of the eye tracking measures as revealed by independent samples *t-*tests (all *p* ≥ 0.111; for more detailed statistics, see Table 3). *BF_10_* ranged from 0.376 to 0.897 (Table 3). Although none of the *BF_10_* values were indicative of substantial support for the null hypothesis, they were nevertheless all below 1. This demonstrates that support for the null hypothesis had a tendency to be stronger than the alternative hypothesis.

**Table 3:** Eye-tracking measures between the ASD and TD groups.

Measurement	*Mean (SD) ASD*	*Mean (SD) TD*	*t_(34)_*	*p_uncorr_*	*p_corr_*	*BF10*
*Saccade velocity (pixels/ms)*	4.11 (0.42)	4.26 (0.65)	0.87	0.388	1	0.433
*Saccade frequency (n/min)*	26.63 (12.87)	36.32 (15.28)	1.63	0.111	1	0.897
*Standard-to-standard saccade (%)*	10.09 (6.60)	11.36 (5.33)	0.63	0.532	1	0.376
*Comparison-to-comparison saccade (%)*	14.83 (6.65)	13.03 (5.08)	0.92	0.365	1	0.447
*Standard-to-comparison saccade (%)*	6.57 (4.28)	8.84 (4.12)	1.62	0.114	1	0.886
*Comparison-to-standard saccade (%)*	7.30 (3.86)	8.91 (4.18)	1.20	0.237	1	0.564
*Other type of saccade (%)*	61.22 (14.77)	57.88 (12.31)	0.74	0.466	1	0.398
*Time spent fixating on standard (%)*	35.72 (12.46)	32.97 (7.62)	0.80	0.430	1	0.413
*Time spent fixating on comparison (%)*	40.58 (13.43)	46.77 (10.89)	1.52	0.138	1	0.782
*Completion time (s)*	61.25 (20.52)	67.47 (27.42)	0.77	0.447	1	0.405

*p_corr_-values reported in column 6 were corrected for multiple correlations using the Bonferroni method*.

Although we could not find any differences between group means, the correlation analyses did yield important results (Table 4; Figures 2B,C). Illusion strength increased as participants made more saccades between different locations within the standard stimulus (*r_(34)_* = 0.46, *p* = 0.005, *BF_10_* = 9.236) (Figure 2B) as well as toward the standard stimulus from the comparison stimulus (*r_(34)_* = 0.48, *p* = 0.003, *BF_10_* = 12.869) (Figure 2C). None of the other correlations were significant (all *p* ≥ 0.111), including *time spent fixating on the standard stimulus* (*p* = 0.430) (Table 4). Thus, illusion strength increased as participants actively made more saccades within and toward the standard stimulus. Simply viewing this same stimulus passively, as indexed by time spent fixating on it, did not increase illusion strength.

**Table 4:** Correlations between illusion strength and different eye-tracking measures.

Correlated with illusion strength	*r_(34)_*	*p_uncorr_*	*p_corr_*	*BF10*
*Saccade velocity (pixels/ms)*	0.24	0.156	1	0.272
*Saccade frequency (n/min)*	0.13	0.452	1	0.547
*Standard-to-standard saccade (%)*	0.46	0.005	*0.049	9.236
*Comparison-to-comparison saccade (%)*	-0.25	0.147	1	0.569
*Standard-to-comparison saccade (%)*	0.39	0.018	0.181	3.029
*Comparison-to-standard saccade (%)*	0.48	0.003	*0.034	12.869
*Other type of saccade (%)*	-0.36	0.030	0.305	1.971
*Time spent fixating on standard (%)*	-0.01	0.972	1	0.208
*Time spent fixating on comparison (%)*	0.15	0.393	1	0.295
*Completion time (s)*	-0.08	0.630	1	0.232

*p_corr_-values reported in column 4 were corrected for multiple correlations using the Bonferroni method. Asterisks (^∗^) denote significance after corrections for multiple correlations were made using this method (*p_*corr*_* < 0.05)*.

Linear regression analyses comparing the slopes between the TD and ASD groups revealed that these correlations seemed to be driven primarily by the ASD group. Specifically, the slopes between the two groups differed for saccades between different locations within the standard stimulus (*F_(1,32)_* = 8.87, *p* = 0.006; ASD group: *r_(16)_* = 0.68, *p* = 0.002, *BF_10_* = 27.281; TD group: *r_(16)_* = -0.09, *p* = 0.735, *BF_10_* = 0.307) (Figure 2B) and for saccades toward the standard stimulus from the comparison stimulus (*F_(1,32)_* = 5.07, *p* = 0.031; ASD group: *r_(16)_* = 0.60, *p* = 0.008, *BF_10_* = 7.723; TD group: *r_(16)_* = 0.22, *p* = 0.378, *BF_10_* = 0.418) (Figure 2C).

In addition to the aforementioned correlations in the overall sample described above, Bayesian statistics also provided substantial support for increases in illusion strength as participants made more saccades toward the comparison stimulus from the standard one (*BF_10_* = 3.029). The Bayesian analyses further revealed substantial support for the null relative to the alternative hypotheses when illusion strength was correlated with *saccade frequency* (*BF_10_* = 0.272), *time spent fixating on the standard stimulus* (*BF_10_* = 0.208), *time spent fixating on the comparison stimulus* (*BF_10_* = 0.295), and *completion times* (*BF_10_* = 0.232).

**FIGURE 3:**
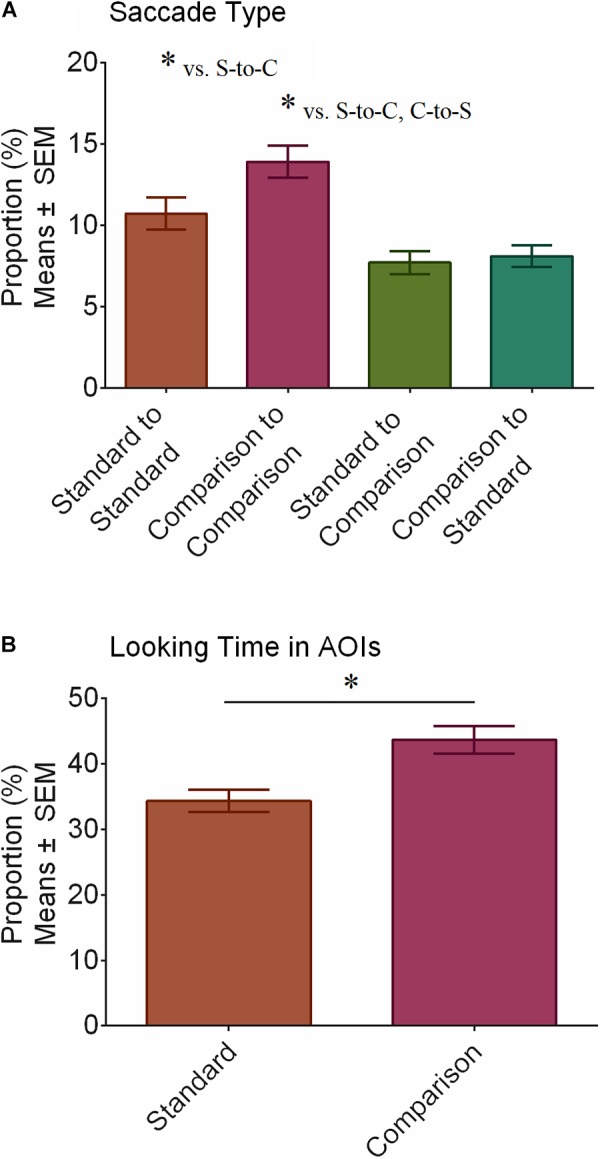
Proportion of saccade types made and time spent looking at AOIs. Participants tended to make more saccades within than between the stimuli as denoted by the asterisks (^∗^) in panel **A**. Looking times were longer for the comparison compared to the standard stimulus, as shown in panel **B**. Asterisks (^∗^) denote significant differences after correcting for multiple comparisons using the Bonferroni method (*p* < 0.05). S-to-C refers to standard-to-comparison saccades and C-to-S refers to comparison-to-standard saccades.

We performed ANOVA with Saccade Type (*standard-to-standard* vs. *comparison-to-standard* vs. *standard-to-comparison* vs. *comparison-to-standard*) and Group (ASD vs. TD) to further explore scan patterns in the Shepard illusion and how they might differ between groups. There was a main effect of Saccade Type (*F_(3,102)_* = 15.17, *p* < 0.001) but not of Group (*F_(1,34)_* = 0.54, *p* = 0.466). The Saccade Type × Group interaction was not significant (*F_(3,102)_* = 1.51, *p* = 0.216). Figure 3A shows the main effect of Saccade Type. *t*-tests, corrected for multiple comparisons using the Bonferroni method, revealed that participants tended to make more saccades within than between stimuli. Specifically, they made more *standard-to-standard* than *standard-to-comparison* saccades (*p* = 0.035, *BF_10_* = 6.70) and more *comparison-to-comparison* saccades than both *standard-to-comparison* (*p* < 0.001, *BF_10_* = 5,450.12) and *comparison-to-standard* (*p* < 0.001, *BF_10_* = 975.66) saccades. All other pairwise comparisons were not significant (*p* ≥ 0.059). Bayesian analyses further revealed substantial support for an increase in *standard-to-standard* relative to *comparison-to-standard* saccades (*BF_10_* = 4.29) and substantial support against differences between *standard-to-comparison* and *comparison-to-standard* saccades (*BF_10_* = 0.32). Bayesian testing for differences (or lack of) between *standard-to-standard* and *comparison-to-comparison* saccades was inconclusive (*BF_10_* = 2.08).

We also performed ANOVA with AOI Looking Time (*standard* vs. *comparison*) and Group (ASD vs. TD). There was a main effect of AOI Looking Time (*F_(1,34)_* = 8.64, *p* = 0.006), whereby participants spent more time looking at the *comparison* than the *standard* stimulus (Figure 3B), no main effect of Group (*F_(1,34)_* = 1.98, *p* = 0.168), and no AOI Looking Time × Group interaction (*F_(1,34)_* = 0.71, *p* = 0.404).

## Discussion

We measured the strength of the Shepard illusion in ASD and TD children and tested if different eye-tracking measurements could predict group differences in illusion strength. We hypothesized that children with ASD would exhibit reduced illusion strength. Replicating previous work in older samples, this hypothesis was confirmed. We also hypothesized that the reduced susceptibility in ASD would be explained by differences in eye-movements. This hypothesis was refuted. None of the eye-tracking measures yielded mean differences between groups, which rules out the possibility that abilities to shift attention (as indexed by *saccade velocity* and *saccade frequency*), attended location (as indexed by the remaining eye tracking measures), and task engagement (as indexed by *completion times*) could explain the mean group difference in illusion strength. However, actively scanning the standard stimulus, as indexed by saccadic movements, enhanced illusion strength more strongly in the ASD than the TD group. Thus, although both groups actively attended to the standard stimulus to the same degree, the ASD group would have perhaps experienced the illusion to the same degrees as the TD group had they actively scanned this stimulus even more than they (and the TD group) did.

In addition, performance on the control matching tasks did not reveal any group differences, ruling out the possibility that the group difference in illusion strength was due to visual discrimination abilities. Moreover, both the ASD and TD children were matched for gender, age, and raw RPM scores, ruling out the possibility that the group difference in Shepard illusion strength was due to gender, chronological age, or non-verbal cognitive abilities. By the process of elimination, we are left to conclude that the two groups integrate similar visual information differently, which ultimately leads to differences in illusion strength. As we discuss below, this processing likely relates to specific kinds of high-level perceptual mechanisms. Another important finding was the presence of greater illusion strength in participants who actively directed their attention most to the standard stimulus.

### Earlier Research on the Shepard Illusion in ASD

The presence of group differences in the strength of the Shepard illusion is consistent with earlier work (Mitchell et al., 2010; Chouinard et al., 2016). We have confirmed in a sample of ASD children the same findings that Mitchell et al. (2010) reported in an older sample of ASD participants between the ages of 12 and 29 years (*M* = 21.1, *SD* = 5.0). Namely, the Shepard illusion was reduced in ASD relative to TD individuals. Our results are also consistent with a previous study that demonstrated a reduction in the strength to the Shepard illusion as a function of autistic traits in the TD adult population (Chouinard et al., 2016). Interestingly, Chouinard et al. (2016) reported that the subscale of the AQ that correlated most strongly with the illusion was the Imagination subscale (*r* = -0.23; i.e., the illusion decreased with levels of imagination given that imagination is restricted in ASD), while the Attention to Detail subscale correlated the least (*r* = -0.06). In light of these findings, Chouinard et al. (2016) argued for the importance of high-level integration mechanisms. Indeed, it is known that the illusion becomes stronger when multiple contextual elements are added to the display. For example, adding legs to the parallelograms to make them look like tabletops strengthens the illusion (Mitchell et al., 2010). Also, the incorporation of additional texture gradients, such as a wooden grain, makes the apparent differences in tabletops even more pronounced (Tyler, 2011).

### The Relevance of the Eye-Tracking Measurements

None of the eye-tracking measures yielded group differences. This has important implications. First, it suggests that abilities to move the eyes and shift attention between different parts of the display were intact in the ASD group. It is conceivable that both *saccade velocities* and *saccade frequencies* would be reduced in the ASD relative to the TD group if this were not the case (Schmitt et al., 2014). Second, it suggests that children with ASD directed their attention to different parts of the visual scene in a similar manner as the TD children. In other words, having an ASD did not determine where a child looked in the visual scene. Together, we can rule out that the group difference in illusion strength was due to abilities in shifting attention and where the children were allocating their attention within the scene.

The reported correlations between the strength of the Shepard illusion and the proportion of saccades made within and toward the standard stimulus are also informative (Figures 2B,C). They demonstrate that the children who made more saccades within and toward the standard stimulus experienced the illusion more strongly. Namely, participants who explored this stimulus more were less veridical in their perceptual experience. Perhaps it is the case that the children who explored the standard more found the percept more interesting, causing the illusion to be stronger, while those who focused more attention elsewhere were less interested. Alternatively, it could be the case that illusion strength causes one to explore the standard more because the illusion itself grabs a child’s attention. Namely, children who experience the illusion more strongly will be more interested in the standard than children who experience the illusion less. Either way, our findings indicate that having an ASD did not predict the degree to which a child would focus their gaze on the standard stimulus. The group means were not statistically different.

However, linear regression analyses comparing the slope of these correlations between the ASD and TD groups revealed that these effects were more prominent in the former group. This is illustrated by the green (ASD children) and blue (TD children) circles in Figures 2B,C. These findings are more difficult to interpret. Perhaps the effects of attending less to the standard stimulus is exacerbated in ASD relative to TD individuals. Alternatively, the differences in slopes could be driven by greater variability in illusion strength in the former group and a ceiling effect in the latter. Future experiments can examine these possibilities further. Nonetheless, the findings indicate that the ASD group might benefit more from actively moving their eyes within and toward the standard stimulus. Strategies that may aid ASD individuals to see this illusion more strongly could have them make more directed eye movements.

It was also interesting to see that the number of saccades made within and toward the standard stimulus predicted illusion strength, while looking time to the same stimulus did not. We believe that this is because the former measurement may reflect more active scrutiny of the stimulus than the latter. Processing the contextual effects provided by the standard stimulus may require an active exploration to different parts of the stimulus rather than maintaining fixation on only a few segments for a prolonged period of time. This finding contradicts an earlier finding from our lab demonstrating a reduction in the strength of the vertical-horizontal illusion when participants were tasked to move their eyes more across the display (Chouinard et al., 2017). We reconcile this discrepancy by suggesting that the vertical-horizontal illusion may depend more on low-level visual processing, whereby greater retinal stability facilitates the processing of perceptual effects. In contrast, the Shepard illusion may depend more on higher-level visual processing in which the registration of multiple contextual elements in different spatial locations of the scene is important in driving the illusion. Indeed, there is considerable evidence to suggest that the Shepard illusion is highly dependent on high-level cognitive processing (Chouinard et al., 2016) and may therefore require more active exploration.

A final note should be raised about null inferences. NHST is not designed to do this – it is only designed to reject the null hypothesis. Conversely, Bayesian statistics enables one to infer whether or not the null hypothesis is supported in cases when NHST did not reject it. However, the Bayesian factors we report in some of these instances are inconclusive (i.e., above 0.33). One should treat these cases with caution.

### Why Are Children With ASD Less Susceptible to the Shepard Illusion?

The following questions then arise: Why are children with ASD less susceptible to the Shepard illusion and what does this tell us about perceptual styles in ASD? As discussed previously, it cannot be because of abilities in shifting attention (given that *saccade velocities* and *saccade frequencies* were the same between groups), where participants directed their attention (given that the remaining eye-tracking measurements also did not reveal group differences), visual discrimination abilities (given the results of the control tasks), and non-verbal cognitive abilities as well as level of engagement (given that both groups were matched on the RPM and completed the task in the same amount of time). By the process of elimination, we are left to conclude that the perceptual differences in ASD are more likely driven by how information is integrated for creating a perception – not by what information is gathered nor by abilities to gather this information nor by intelligence. In the past, we have offered three possible explanations to explain why the Shepard illusion might be diminished in ASD (Chouinard et al., 2016). We will briefly summarize each one of them.

The first relates to shape processing. Badcock and his colleagues have demonstrated how abilities in shape processing can be substantially different in ASD (Grinter et al., 2010; Almeida et al., 2014). However, we could not demonstrate any evidence for this in our shape matching control task given that both groups performed the task to the same level. Admittedly, our control task is not as sensitive for detecting differences in shape processing as the more sophisticated paradigms with radial frequency patterns used by Badcock and his colleagues. Hence, we believe that shape processing and how this relates to the Shepard illusion could still be a promising avenue for future research.

The second relates to mental rotation. The mechanisms of the Shepard illusion are unknown but it is tempting to infer that they might involve mental rotation. After all, the illusion consists of the same shape oriented in two different ways and it was first described by Shepard (1990), who was a pioneer in mental rotation research. In addition, males are reported to be better than females on mental rotation tasks (Voyer et al., 1995) and it has been argued that ASD could be an extreme manifestation of a male brain (Baron-Cohen, 2002). Hence, it could be the case that the ASD participants in our study were better at mental rotation and could see the parallelogram as having the same dimensions when presented in two different orientations. However, the evidence for enhanced mental rotation abilities in ASD remains inconsistent and controversial (Soulières et al., 2011; Beacher et al., 2012; McGrath et al., 2012) – indicating that further research on mental rotation and the Shepard illusion in ASD is required.

The third relates to the integration of within-contextual properties. Geometric illusions can be grossly lumped into two classes: between-object and within-object illusions (Ben-Shalom and Ganel, 2012). In between-object illusions, such as the well-known Ebbinghaus illusion (Titchener, 1901), the target stimulus is physically separated from the elements in the background that provide context. In contrast, for within-objects illusions, such as the Shepard illusion, it is the various characteristics of the target stimulus itself (e.g., its length and width) that provides context. Our earlier research demonstrates how illusion strength in TD adult populations decreases more strongly as a function of autistic traits for within-object illusions, such as the Shepard illusion, than they do for between-object illusions (Chouinard et al., 2013, 2016).

Most illusions can be considered intermediates between these two classes of illusions, some of which have yielded more inconsistencies with regards to susceptibility in ASD than the Shepard illusion. For example, Ropar and Mitchell (1999), Bölte et al. (2007), and Manning et al. (2017) have demonstrated increases in the strength of the Müller-Lyer illusion in ASD, while a number of studies have not (Happé, 1996; Ropar and Mitchell, 2001; Hoy et al., 2004; Ishida et al., 2009). Likewise, Happé (1996), Bölte et al. (2007), and Ishida et al. (2009) have demonstrated decreases in the strength of the Ponzo illusion in ASD, while a number of studies have not (Ropar and Mitchell, 1999, 2001; Hoy et al., 2004). In addition, Ropar and Mitchell (1999) demonstrated decreases in the vertical-horizontal illusion, which they did not replicate in a later study (Ropar and Mitchell, 2001). Perhaps, these inconsistencies relate to the processing of both between- and within- contextual elements present in these illusions in which the latter but not the former might be affected by ASD.

These inconsistencies further underscore how perceptual styles in ASD might be driven by some kind of high-level integration mechanism yet to be identified as opposed to a more general break-down and/or a non-preference for global integration as posited by several theories reviewed in the Introduction [EPF theory (Mottron et al., 2006), WCC theory (Happé and Frith, 2006), and Bayesian-based explanations (Pellicano and Burr, 2012)], as well as emerging ideas that sensory processing is aberrant in ASD from the earliest stages of sensory processing (Robertson and Baron-Cohen, 2017), including dorsal stream processing (Spencer et al., 2000; Robertson et al., 2012). If these notions were correct then one would expect to see more consistencies across studies examining perceptual processes in ASD – which we consider highly inconsistent. The reduction of illusion strength in the Shepard illusion is one of only a few examples of consistent findings across studies.

### Implications

The findings of the current study converge with other earlier studies demonstrating reduced strength of the Shepard illusion in an older sample with ASD (Mitchell et al., 2010) and as a function of autistic traits in the adult TD population (Chouinard et al., 2016). For the first time, we acquired eye-tracking data as individuals with ASD performed illusion tasks. The use of eye-tracking enabled us to more confidently rule out a number of competing explanations. We conclude that the reasons as to why the Shepard illusion is reduced in children with an ASD are perceptual rather attentional. The reduced strength of the Shepard illusion in ASD does not appear to be driven by how this population allocates attention but rather how they might integrate visual information for the purposes of creating a perceptual experience.

## Author Contributions

PC, IS, and OL conceived and designed the experiments. KR and OL collected the data. All authors contributed to the analysis and writing of the manuscript.

## Conflict of Interest Statement

The authors declare that the research was conducted in the absence of any commercial or financial relationships that could be construed as a potential conflict of interest.
